# Genetic aspects underlying the normocalcemic and hypercalcemic phenotypes of primary hyperparathyroidism

**DOI:** 10.1007/s12020-023-03476-7

**Published:** 2023-08-31

**Authors:** Arianna Viviani, Luciano Colangelo, Bianca Maria Ciminelli, Andrea Novelletto, Chiara Sonato, Marco Occhiuto, Cristiana Cipriani, Daniele Diacinti, Viviana De Martino, Walter Gianni, Jessica Pepe, Salvatore Minisola, Patrizia Malaspina

**Affiliations:** 1 Department of Biology, University of Rome Tor Vergata, Rome, Italy; 2 Department of Clinical, Internal, Anesthesiologic and Cardiovascular Sciences, “Sapienza” University of Rome, Rome, Italy; 3 Department of Radiological Sciences, Oncology and Pathology, “Sapienza” University of Rome, Rome, Italy

**Keywords:** Normocalcemic primary hyperparathyroidism, SNPs, ESR1, VDR, OPG, RANKL

## Abstract

**Purpose:**

Hypercalcemic primary hyperparathyroidism (PHPT) is a common endocrine disorder that has been very well characterized. In contrast, many aspects of normocalcemic primary hyperparathyroidism (NPHPT) such as natural history, organ damage, and management are still matter of debate. In addition, both the pathophysiology and molecular basis of NPHPT are unclear. We investigated whether PHPT and NPHPT patient cohorts share the same pattern of genetic variation in genes known to be involved in calcium and/or bone metabolism.

**Research design and methods:**

Genotyping for 9 single nucleotide polymorphisms (SNPs) was performed by Real-Time PCR (TaqMan assays) on 27 NPHPT and 31 PHPT patients evaluated in a tertiary referral Center. The data of both groups were compared with 54 in house-controls and 503 subjects from the 1000 Genomes Project. All groups were compared for allele/haplotype frequencies, on a single locus, two loci and multi-locus basis.

**Results:**

The NPHPT group differed significantly at SNPs in OPG and ESR1. Also, the NPHPT cohort was peculiar for pairwise associations of genotypes and for the overrepresentation of unusual multilocus genotypes.

**Conclusions:**

Our NPHPT patient set harbored a definitely larger quota of genetic diversity than the other samples. Specific genotypes may help in defining subgroups of NPHPT patients which deserve ad hoc clinical and follow-up studies.

## Introduction

Primary hyperparathyroidism (PHPT) is a common endocrine disorder with the biochemical signature of hypercalcemia and either raised or inappropriately normal serum concentration of parathyroid hormone [1].

The prevalence of hypercalcemic PHPT in the population is rising, due to advances in diagnostics and the introduction of routine accurate measurements of serum levels of calcium and PTH [2]. Indeed, this rise of prevalence has occurred mainly because of the use of serum calcium determination as part of a multichannel screening profile, probably leading to an earlier detection of patients with primary hyperparathyroidism [3]. As a result, this led to a shift in the presentation of hypercalcemic PHPT from a predominantly symptomatic to an asymptomatic disease.

In addition, a new entity called normocalcemic primary hyperparathyroidism (NPHPT) has also been recognized [4]. This latter is characterized by normocalcemia and persistent elevated serum parathyroid hormone values, after excluding conditions determining raised parathyroid hormone levels.

NPHPT has been extensively studied to evaluate if complications of the disease were similar to its hypercalcemic counterpart [5–7]. However, a recent consensus statement acknowledges paucity of data concerning some aspects of the disease, such as pathophysiology, natural history, organ damage and management [3].

The present study was therefore carried out to investigate whether PHPT and NPHPT patient cohorts share the same pattern of genetic variation in genes known to be involved in calcium and/or bone metabolism.

## Materials and methods

We genetically typed 31 patients with hypercalcemic PHPT (2 males and 29 females) and 27 patients with NPHPT (5 males and 22 females) diagnosed at Mineral Metabolism Center of Policlinico Umberto I, "Sapienza" Rome University during the period from September 2017 to October 2021. Initially, each patient underwent a thorough medical history, physical examination, and laboratory exams to exclude the presence of comorbidities and/or medications known to influence the investigated parameters (i.e., steroid therapy or diuretics and lithium salts).

The diagnosis of PHPT was made by conventional laboratory criteria, namely the finding of hypercalcemia and elevated or inappropriately normal serum PTH levels for at least 1 year. Following the international recommendation [1], in case of an increase of PTH levels with both total and ionized calcium in the normal range, before making the diagnosis of NPHPT, we excluded secondary forms of hyperparathyroidism including vitamin D deficiency, renal failure, malabsorption, low calcium intake, hypercalciuria and medications. Biochemical parameters were assayed as previously described [8]; in particular, serum PTH and 25(OH)vitamin D were measured by chemiluminescence-immunoassay (CLIA) with the fully automated LIAISON® analyzer. Intra and inter-assay coefficients of variation were 4.1% and 5.2%, respectively. Since 7% of patients studied (i.e., four patients, two each in the normocalcemic and hyper calcemic group) had serum PTH and total alkaline phosphatase determined outside of our laboratory, data for this parameter are reported as z-scores in respect to the average of normal range.

Each patient had abdominal ultrasound performed by a skilled radiologist. Each ultrasonogram was performed with a low-to-medium frequency (3.5–5 MHz, depending on the physical characteristics of the subject) convex probe and the ultrasound scanner (Esaote MyLab 70 X Vision; Esaote). Ultrasonography was performed in the supine, right and left lateral decubitus positions. The presence, number, and position of stones were evaluated. Renal stones were detected by specific ultrasonographic signs, such as hyperechogeneity and posterior acoustic shadowing. Bone mineral density values were measured by DXA (QDR-4500 Hologic Inc., USA) at the lumbar spine (L1-L4), femoral neck (FN), total hip (TH), and distal radius in all patients.

All patients gave written, informed consent before their inclusion in the study. The investigation was approved by the Institutional Review Board of the Department of Clinical, Internal, Cardiovascular and Anesthesiologic Sciences and then approved by the Ethics Committee of “Sapienza”, University of Rome (protocol number 3040N 73/14). The Research was carried out complying with the World Medical Association Declaration of Helsinki.

### Genotyping

Genomic DNA was extracted from peripheral blood using laboratory standard procedures. We analyzed the 9 SNPs listed in Table S1, more involved in the regulation of calcium homeostasis and bone density. Genotyping was performed by TaqMan allele discrimination assays (Applied Biosystem) according to the manufacturer’s instructions. Genotype was assigned by registering the fluorescence emission from each sample at the VIC and FAM dye wavelengths. The same assays were applied to all in-house controls, i.e., 54 anonymous control DNAs routinely used in the lab (hereafter in-house controls), and additional 106 subjects from Central-South Italy [9], typed only for SNPs in PTH and ESR1.

The above datasets were integrated with genotypes for the 503 subjects of European descent of the 1000 Genomes project [10]. The data slicer available at http://www.ensembl.org/index.html was used to extract genotype data at the relevant and surrounding positions from high coverage sequencing results deposited at http://ftp.sra.ebi.ac.uk/.

### Genetic data encoding

In order to run sparse Principal Component (sPC) analysis genotypes at biallelic loci were encoded by paired variables with 0’s and 1’s indicating the absence or the presence of the reference and alternative allele [11].

Two SNPs at the ESR1 locus (in the order rs9340799-rs2234693), and two at the VDR locus (in the order rs731236-rs7975232) were combined into haplotypes. For the patients, in-house and additional controls, phasing was obtained with Phase2 [12]. For the 1000 Genomes dataset the phasing reported in the sliced VCF files was retained.

These procedures resulted in four observed haplotypes at ESR1 and only three at VDR, that were encoded into four and three binary variables, respectively (Table S1).

### Data analysis

Allele/haplotype frequencies and the testing of Hardy-Weinberg equilibrium were obtained with Arlequin [13] for each group of subjects, separately. All remaining calculations were performed in an R environment. Multidimensional analysis was performed by sPC [14] as implemented in the package “sparsepc” (https://github.com/erichson/spca). sPCA attempts to find sparse weight vectors (loadings), i.e., a weight vector with only a few “active” (nonzero) values. This approach provides better interpretability for the principal components in high dimensional data settings. This is because the principal components are formed as a linear combination of only a few of the original variables. This is a powerful method to analyze differentiation at multiallele systems and takes into account the presence/absence of alleles at each locus. Our dataset then included 17 such variables (Table S1) for all patients. In-house controls and the 1000 Genomes subjects were projected on the same plot using their own genotype vectors and the same loadings.

## Results

### Contrasting features of the normo- and hypercalcemic subgroups

Anthropometric and biochemical parameters of patients in the two groups are reported in Table 1. By definition, both mean total [10.97 vs 9.60] and ionized serum calcium values [1.42 vs 1.26] were significantly higher in hypercalcemic vs normocalcemic patients (*p* < 0.001 in both cases). Furthermore, other biochemical parameters were significantly different between the two groups. Among these, mean z-score PTH values were significantly raised in the hypercalcemic group (*p* = 0.037), as well as mean ALP z-score values (*p* < 0.001) Finally, the prevalence of lithiasis was higher in hypercalcemic patients, with macrolithiasis affecting one third of hypercalcemic patients but none of the normocalcemic ones (X-squared = 8.3849, df = 1, *p* value < 0.001).

**Table 1:** Demographic parameters and clinical, biochemical and radiological values

Variables and abbreviations	Units		All patients *n* = 58	NPHPT *n* = 27	PHPT *n* = 31	NHPTH vs PHPT *p* value
M:F			7:51	5:22	2:29	
Age	Years	Mean	64.12	63.37	64.77	
		s.d.	8.47	6.26	10.10	
Age at menopause (AAM)	Years	Mean	48.28	47.64	48.79	
		s.d.	5.73	6.43	5.18	
Years Since Menopause (YSM)	Years	Mean	16.36	16.41	16.32	
		s.d.	9.86	9.27	10.48	
Body Mass Index (BMI)	kg/m^2^	Mean	24.36	23.98	24.69	
		s.d.	4.17	3.58	4.65	
Serum Calcium (sCa)	mg/dL	Mean	10.33	9.60	10.97	<0.001
		s.d.	0.81	0.37	0.49	
Ionized Calcium (Ca^++^)	mmol/L	Mean	1.35	1.26	1.42	<0.001
		s.d.	0.10	0.03	0.09	
24 h urinary calcium (uCa/24 h)	mg	Mean	228.80	177.70	273.30	<0.001
		s.d.	106.17	67.96	114.08	
Serum Phosphate (sP)	mg/dL	Mean	3.09	3.38	2.85	
		s.d.	0.53	0.39	0.51	
25(OH)vitamin D (s25(OH)D	ng/mL	Mean	36.78	39.13	34.74	
		s.d.	12.77	15.87	9.07	
Parathyroid Hormone (PTH z-score)	s.d. units	Mean	5.46	4.31	6.47	0.0372
		s.d.	4.13	2.00	5.17	
Serum Creatinine (sCr)	mg/dL	Mean	0.84	0.86	0.83	
		s.d.	0.14	0.14	0.15	
Alkaline phosphatase z-score (ALP)	s.d. units	Mean	0.18	−0.32	0.62	<0.001
		s.d.	1.07	0.91	1.02	
Calcium/Creatinine clearance ratio (CCCR)		Mean	0.02	0.02	0.02	
		s.d.	0.01	0.01	0.01	
T-score Lumbar		Mean	−2.14	−1.83	−2.42	
		s.d.	1.41	1.52	1.27	
T-score Neck		Mean	−2.13	−2.02	−2.23	
		s.d.	0.88	0.85	0.91	
T-score Total		Mean	−1.76	−1.60	−1.91	
		s.d.	0.92	0.95	0.88	
Vertebral fractures		%	36.21	37.04	35.48	
Osteoporosis		%	75.86	74.07	77.42	
Lithiasis		%	36.21	22.22	48.39	0.0728
Lithiasis (macrolithiasis)		%	17.24	0.0	32.26	0.0038

Table 1: mean and standard deviation (s.d.) of parameters in patients with Normocalcemic Primary Hyperparathyroidism (NPHPT) and patients with Primary Hyperparathyroidism (PHPT)

### Genetic diversity among patients

In the overall patient cohort, allele or haplotype frequencies (Table 2 top) matched, in general, those reported for the European population and represented among 503 subjects typed in the frame of the 1000 Genomes project. Our in-house controls, with a sample size comparable to the patients, displayed similar frequencies.

**Table 2:** Allele, haplotype (top) and genotype (bottom) frequencies in the population groups considered




*SNP* Single Nucleotide Polymorphism, *NPHPT* normocalcemic primary hyperparathyroidism, *PHPT* hypercalcemic primary hyperparathyroidism, *Freq*. frequency, *s.e.* standard error, *HW* Hardy-Weinberg equilibrium test, *OPG* osteoprotegerin, *RANK-L* receptor activator of nuclear factor kB ligand, *PTH* parathyroid hormone, *CASR* calcium sensing receptor, *COL1A1* collagen type I alpha 1 chain, *ESR1* estrogen receptor 1, *VDR* vitamin D receptor

In this work we focused on the NPHPT group. We then asked whether the genotype distributions could distinguish this group from the other samples, thus identifying risk loci for NPHPT (Table 2 bottom). In fact, at rs6256 (PTH) the normocalcemic group differed significantly from our in-house controls (*p* = 0.044), showing an excess of (T/T) homozygotes, i.e., a proportion higher than all other control groups.

Also, the normocalcemic group differed significantly at rs2073618 (OPG) from both the hypercalcemic group (*p* = 0.02) and the 1000 Genomes group (*p* = 0.04). These patients displayed a definite excess of OPG(G/C) heterozygotes (H.W. test *p* = 0.007). At rs9340799-rs2234693 (ESR1), the two patient subgroups differed significantly (*p* = 0.02). The test of Hardy-Weinberg equilibrium revealed a significant departure in the normocalcemic group. Forty percent and 33% of AT/AT and GC/GC homozygotes were observed, respectively, as contrasted to 33% and 9% in the 1000 Genomes group (*p* < 0.001), 20% and 20% in in-house controls, and 25% and 14% in additional controls (*p* = 0.044), respectively.

When examining pairwise interactions between loci in the NPHPT subgroup (Table S3), significant unbalanced joint distributions were found for ESR1 (rs9340799-rs2234693)-RANKL (rs9525641) and VDR (rs731236-rs7975232)-OPG (rs2073618). In particular, the genotype ESR1(GC/GC)-RANKL(C/C) was remarkably enriched among the normocalcemic patients (22%) as compared to both the 1000 Genomes group (1.8%) and in-house controls (5.6%) (*p* < 0.001 and n.s., respectively). Moreover, we observed an enrichment (26%) of VDR(AA/AA and AA/AC)-OPG(G/C), which contrasted with the findings in the 1000 Genomes group (9%) and in-house controls (11%)(*p* = 0.012 and n.s., respectively).

**Fig. 1:**
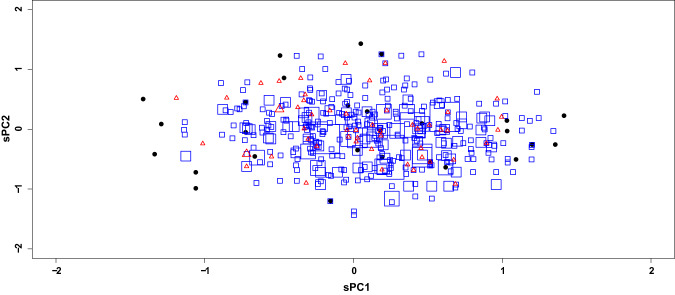
Plot of the 27 NPHPT patients in the space of sPC’s 1 and 2 (black dots). The 1000 Genomes EUR subjects (*n* = 503, blue squares) and the 54 in-house controls (red triangles) were projected on the same space (square and triangle size proportional to the n. of observations)

When sPCA was used to represent the overall genetic diversity of the normocalcemic group (Fig. 1), four alleles/haplotypes contributed mostly (loadings < −0.40 or >0.40) to PC1 (27% of total variance), i.e., ESR1(GC), ESR1(AT), VDR(GA) and RANKL(T). PC2 (16% of total variance) was mainly contributed by PTH(T), COL1A1(T) and VDR(AA).

In order to check whether the genetic diversity represented among our patients could be considered a random sample as compared with subjects with a comparable continental ancestry background, we projected on the same space the 54 in-house controls and the 503 subjects of the 1000 Genomes project. The plot displayed largely overlapping clouds of points, but the genotypes of some of our patients were not matched by any other, despite a 20-fold larger sample size. Notably, this occurred for both extremely high and low PC1 values, and for the highest PC2 values.

The genotypes found only among NPHPT patients included ESR1(GC/GC);VDR(GA/AC);RANKL(C/C) at the left-end of the plot and ESR1(AT/AT);VDR(AA/AC or AA/AA) at the right-end of the plot. This latter subgroup was strongly enriched in OPG(G/C). Nine-SNP genotypes producing PC1 values lower than −1 were strongly enriched in NPHPT patients (*p* < 0.001 and *p* = 0.07 when compared to 1000 Genomes and in-house controls, respectively). The same was true for genotypes producing PC1 values higher than +1 (*p* < 0.001 and *p* = 0.016, respectively).

In summary, our NPHPT patient set harbored a definitely larger quota of genetic diversity than the other samples.

## Discussion

We analyzed nine single nucleotide polymorphisms in two groups of patients with hypercalcemic and normocalcemic primary hyperparathyroidism, very well characterized from a biochemical point of view. These two groups were compared with the results obtained from in-house controls and 503 subjects of European descent.

Concerning the normocalcemic group, which constituted 46.5% of the whole patient series, we analyzed genetic data on a single locus basis, as well as considering two-loci genotype distributions and a multidimensional genotype representation. We found instances of genotype imbalances at individual loci.

Our results suggest that the rs9340799-rs2234693 genotype (GC/GC) at ESR1 is associated to NPHPT. Indeed, primary hyperparathyroidism is often diagnosed in women, in the first decade after menopause, consistent with the known skeletal action of estrogen that neutralizes the hypercalcemic effects of excess PTH in bone [15]. However, it is unclear whether this genotype simply delays the transit from the normo- to the hypercalcemic state or marks subjects who remain normo-calcemic. Ad-hoc follow-up studies will be needed to clarify this point. At any rate, ESR1 can be considered for an initial genetic characterization of PHPT patients. In this context, it should be emphasized that the natural history of NPHPT is still unclear. Eastell et al. [16] performed a 5-years retrospective evaluation of a cohort of NPHPT patients. They concluded that NPHPT may be a mild form of PHPT.

As to the higher incidence of the rs6256(T/T) genotype at PTH in normocalcemic patients, the significance is elusive. At this site, the alternative (T) variant generates a premature stop codon p.(Arg83*) that determines a truncation of the mature PTH peptide to 52 amino acids, causing impairment of translocation across the endoplasmic reticulum, cleavage of pro-PTH and secretion of PTH [17, 18]. No specific function has been attributed to the portion of the secreted PTH polypeptide downstream to pos. 83, so far. Instead, the two NPHPT patients with the T/T genotype displayed significantly higher PTH levels and calcium excretion, while lower phosphorus values as compared to the alternative genotypes (Kruskal-Wallis *p* = 0.028, 0.037, and 0.037, respectively). Our results are at odds with the findings in ref. [19], and point to a potentiation of PTH activity when the C-terminal portion is present [15]. Further experimental models should evaluate if this polymorphism could have implications for tissue-specific biological actions of PTH.

Also, the NPHPT cohort was peculiar for pairwise associations of genotypes, pointing towards gene effect interactions that are associated to NPHPT. These associations in the NPHPT cohort raise some hypotheses. In fact, significantly unbalanced distributions of two-loci genotypes were observed for ESR1-RANKL, and VDR-OPG. It is well established how the receptor activator of nuclear factor-κB (RANK), RANK ligand (RANKL), and its decoy receptor osteoprotegerin (OPG) play key roles in regulating bone turnover [20]. Previous data showed as baseline serum concentrations of OPG and RANKL were higher in PHPT patients than in healthy controls, whilst the OPG/RANKL-F ratio was lower [21]. In addition, the latter authors also reported the absence of changes of serum osteoprotegerin values following parathyroidectomy.

Hence, even though no single SNP typed in our cohort was able to explain a relevant proportion of the overall diversity, it may be plausible that some genotype associations at two or three loci (implicated in calcium homeostasis) may guide in working out a genetic contribution to different phenotypes in PHPT or NPHPT.

Our multivariate analysis, aimed at condensing the genotype heterogeneity of the NPHPT cohort, revealed overrepresentation of unusual multilocus genotypes. As compared to previous works in the literature [19, 22], this method can potentially capture the combined effect of multiple alleles/haplotypes even when the contribution of each of them is subtle. These genotypes may help in defining subgroups of NPHPT patients which deserve ad hoc clinical and follow-up studies to tackle the question of why “some patients develop skeletal, renal or other complications while others do not [15]”. To improve genetic identification of risk factors, further polymorphisms in genes potentially involved in clinical manifestations should be analyzed. Among them, particularly relevant could be additional SNPs in genes encoding for receptors of calcium (CASR) [23, 24], PTH (PTHPR1 and PTHPR2) and calcitonin (CalcR).

## Conclusion

This is the first report exploring the genetic aspects underlying the two phenotypes of primary hyperparathyroidism, i.e., the features of the normocalcemic and hypercalcemic cohorts. Long-term longitudinal studies are needed to evaluate if specific polymorphisms could be able to target those individuals transitioning from normocalcemic to hypercalcemic state.

### Supplementary information

SupplementaryTables

## Data Availability

The data that supported the findings of this study are available from the corresponding author upon reasonable request.
